# Lysosomal protease cathepsin D; a new driver of apoptosis during acute kidney injury

**DOI:** 10.1038/srep27112

**Published:** 2016-06-07

**Authors:** Pasquale Cocchiaro, Christopher Fox, Nicholas W. Tregidgo, Rachel Howarth, Katrina M. Wood, Gerhard R. Situmorang, Luigi M. Pavone, Neil S. Sheerin, Anna Moles

**Affiliations:** 1 Fibrosis Research Group, Institute of Cellular Medicine, Newcastle University, Newcastle Upon Tyne, UK; 2 Department of Molecular Medicine and Medical Biotechnology, University of Naples, Federico II, Italy; 3 Department of Cellular Pathology, Royal Victoria Infirmary, Newcastle Upon Tyne, UK; 4 Urology Department, Cipto Mangunkusumo National Referral Hospital/Faculty of Medicine, University of Indonesia, Jakarta, Indonesia.

## Abstract

Acute kidney injury (AKI) is an abrupt reduction in kidney function caused by different pathological processes. It is associated with a significant morbidity and mortality in the acute phase and an increased risk of developing End Stage Renal Disease. Despite the progress in the management of the disease, mortality rates in the last five decades remain unchanged at around 50%. Therefore there is an urgent need to find new therapeutic strategies to treat AKI. Lysosomal proteases, particularly Cathepsin D (CtsD), play multiple roles in apoptosis however, their role in AKI is still unknown. Here we describe a novel role for CtsD in AKI. CtsD expression was upregulated in damaged tubular cells in nephrotoxic and ischemia reperfusion (IRI) induced AKI. CtsD inhibition using Pepstatin A led to an improvement in kidney function, a reduction in apoptosis and a decrease in tubular cell damage in kidneys with nephrotoxic or IRI induced AKI. Pepstatin A treatment slowed interstitial fibrosis progression following IRI induced AKI. Renal transplant biopsies with acute tubular necrosis demonstrated high levels of CtsD in damaged tubular cells. These results support a role for CtsD in apoptosis during AKI opening new avenues for the treatment of AKI by targeting lysosomal proteases.

Acute Kidney Injury (AKI), as defined by the Acute Kidney Injury Network, is an abrupt (within 48 hours) reduction in kidney function as measured by an increase in serum creatinine or a reduction in urine output^1^. AKI is common affecting 3–18% of all hospitalized patients^2^,^3^. It is associated with high morbidity and mortality (30–70%) and can have long term consequences increasing the risk of developing Chronic Kidney Disease (CKD)^4^.

Depending on its cause, AKI can be classified as prerenal (decreased blow flow), intrinsic (direct damage) or postrenal (urinary tract obstruction). Despite the heterogeneity of causes the subsequent response to injury involves similar pathways including apoptosis and necrosis. Ischemia^5^ and nephrotoxicity^6^ are key drivers of the cellular injury, which leads to the functional and structural changes resulting in a decline in renal function. There is no specific therapy to treat AKI and current treatments focus on the management of the underlying cause, however, in some cases renal replacement therapy may also be required^7^. Therefore, a better understanding of the cellular processes driving cellular injury during AKI is essential to find new therapeutic targets that could preserve renal function.

Renal epithelial tubular cells, particularly proximal tubular cells, are highly vulnerable to cell death by toxic or ischemic injury. They are exposed to high levels of circulating toxics due to its reabsorbing and concentrating role of the glomerular filtrate. In addition, they have low glycolytic capacity to produce ATP, which will compromise their survival under ischemic conditions and impair oxidative metabolism. Both apoptosis and necrosis coexist during AKI. It is classically thought that the severity of the injury and the availability of ATP will determine the type of the cell death occurring. Apoptosis is a tightly controlled process^8^ regulated by the activity of caspases, which are the main initiators and effectors of apoptosis. The mechanism of caspase cascade activation defines the type of apoptosis: intrinsic (mitochondria dependent) or extrinsic (death receptor mediated).

Lysosomes contribute to necrosis if complete lysosomal rupture occurs but also can drive apoptosis due to the release of lysosomal hydrolases, cathepsins, into the cytosol as a consequence of lysosomal membrane permeabilization (LMP)^9^,^10^. Cathepsins have been widely implicated in apoptosis^11^. They are release into the cytosol as active enzymes where they can interact with a variety of substrates (Bcl-2 family proteins Bid, Bcl-2, Bcl-XL, and Mcl-1, XIAP, caspases-2 and -8, phospholipase A2 (PLA2) and sphingosine kinase-1)^12^,^13^ contributing to caspase dependent and independent apoptosis with or without mitochondrial involvement^14^,^15^.

Despite the well characterized role of cathepsins in apoptosis and the importance of apoptosis in AKI, the contribution of cathepsins to AKI is still unknown. The aim of this study was to analyse the role of cathepsin D (CtsD) in AKI. CtsD was upregulated in two mice models of AKI. Pharmacological inhibition of CtsD reduced functional and histological injury as well as the level of apoptosis induced in both models of disease. Inhibition of CtsD also diminished the degree of interstitial fibrosis that developed after IRI induced AKI. These results suggest an important role for CtsD in the development of AKI and subsequent complications.

## Results

### Cathepsin D expression is upregulated in folic acid induced nephrotoxic AKI

**Figure 1:**
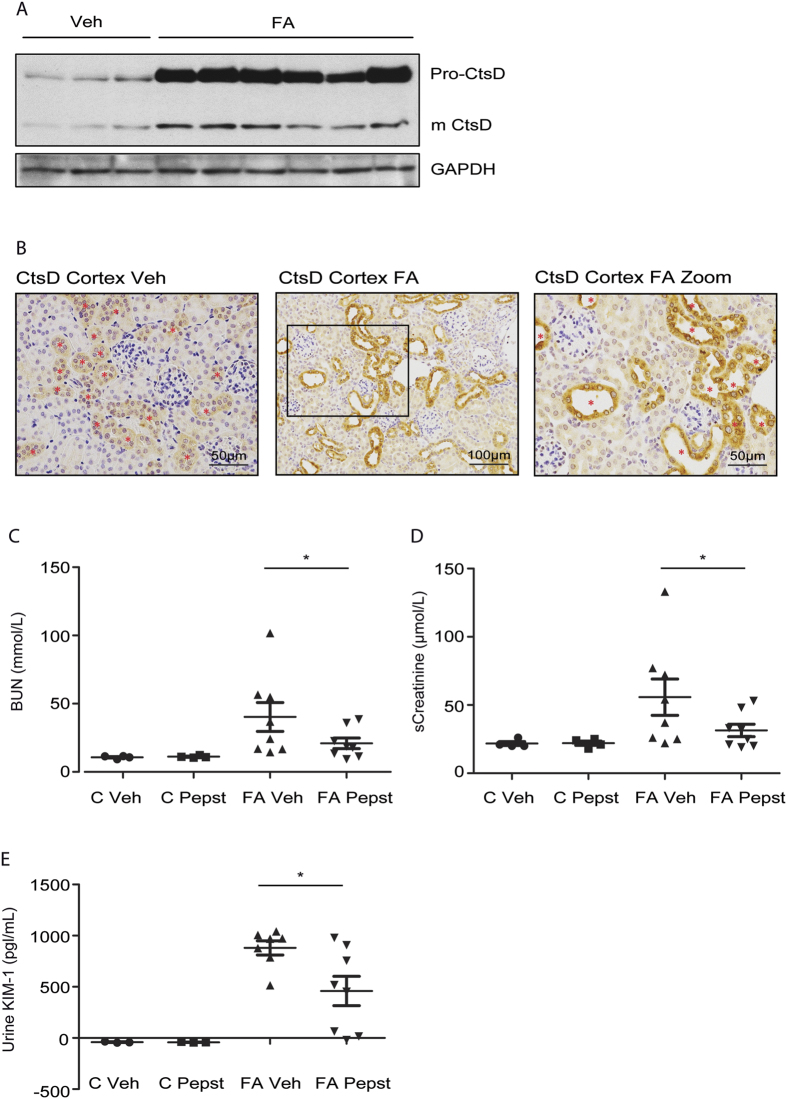
CtsD inhibition improves kidney function in FA nephrotoxic induced AKI. Western Blot of pro- and mature CtsD and GAPDH in kidney lysates. **(A)** Representative pictures and magnified area of CtsD cortical staining in control and 48 hours FA treated kidneys. Red stars * point CtsD expressing cells**. (B)** BUN **(C)**, serum creatinine **(D)**, urine KIM-1 ELISA **(E)** in control and 48 hours FA vehicle and Pepstatin A treated kidneys. Animals were treated with vehicle or Pepstatin A (20 mg/Kg) 45 minutes before and 24 hours post-FA. N = 8, repeated measures of t-test, *P ≤ 0.05 or **P ≤ 0.01.

Nephrotoxic acute kidney injury can happen due to a wide variety of commonly used drugs^6^. Intrinsic damage into the kidney tissue will lead to cell death contributing to the decline in kidney function. We first analysed CtsD expression in folic acid (FA) induced AKI. Administration of high doses of FA induces acute tubular necrosis by formation of crystals mainly within the cortical area^16^. Pro- and mature forms of CtsD were increased after 48 hours of FA injection (Fig. 1A). CtsD staining of kidney cortex confirmed this increase in damaged tubular epithelial cells (Fig. 1B). In agreement with Fig. 1A, CtsD was also detected in some tubular cells of vehicle treated animals despite its expression was much less than in injured kidneys (Fig 1B, Suppl. Fig. S1A,B). Dual immunofluorescence for markers of proximal (Aquaporin-1) or distal (thiazide-sensitive NaCl co-transporter or NCC) tubular epithelial cells and CtsD, localized CtsD mainly in distal tubular cells, however, some expression was also detected in proximal tubular cells in vehicle and 48 hours FA injured kidney tissues (Suppl. Fig. S1A,B). Therefore CtsD could contribute to tubular injury during FA induced AKI.

### Pepstatin A administration improves kidney function after folic acid induced nephrotoxic injury

**Figure 2:**
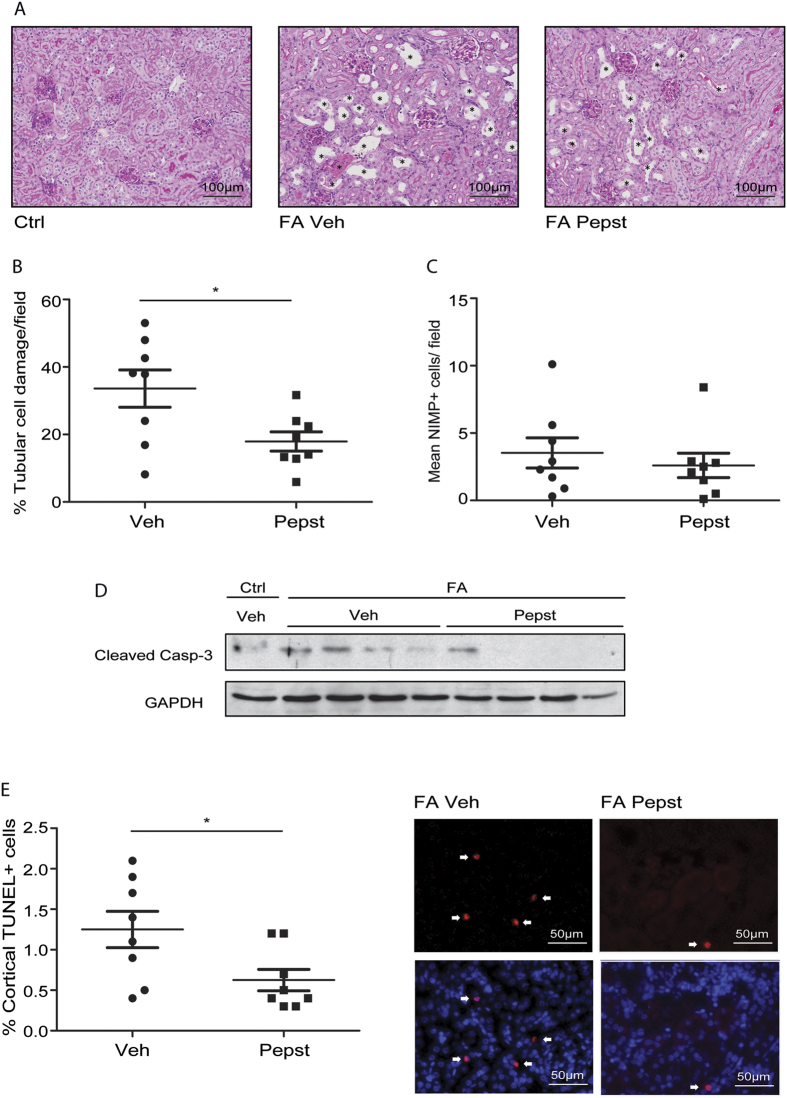
CtsD inhibition reduces tubular cell injury and apoptosis in FA nephrotoxic induced AKI. Representative PAS pictures, damaged cells pointed with a black star *. **(A)** Percentage of tubular cell injury in cortex **(B)** as assessed by tubular dilatation, epithelial flattening and loss of brush border in FA vehicle or Pepstatin A treated kidneys. Average number of NIMP^+^ cells per field in FA vehicle or Pepstatin A treated kidneys. **(C)** Cleaved caspase-3 and GAPDH WB **(D)** of control vehicle and FA vehicle or Pepstatin A treated kidneys. Percentage of cortical TUNEL positive cells versus total cells and representative TUNEL only or DAPI merged pictures **(E)** in FA vehicle or Pepstatin A treated kidneys. White arrows point to TUNEL^+^ cells. Animals were treated with vehicle or Pepstatin A (20 mg/Kg) 45 minutes before and 24 hours post-FA. N = 8, t-test, *P ≤ 0.05 or **P ≤ 0.01.

To study the role of CtsD in nephrotoxic induced AKI we administered the CtsD inhibitor, Pepstatin A, 45 minutes and 24 hours after FA injection. Kidney function was assessed 48 hours post-FA administration by blood urea nitrogen (BUN) and serum creatinine. As expected both markers were increased by FA (Fig. 1C,D). Pepstatin A treatment significantly reduced BUN and serum creatinine levels in animals undergoing AKI (Fig. 1C,D) showing improved kidney function. KIM-1, a tubular injury biomarker^17^, was measured in urine with similar results (Fig. 1E). Histological injury caused by FA treatment was reduced by 40% in animals treated with Pepstatin A (Fig. 2A,B). According to our results inhibition of CtsD leads to an improvement in kidney function and a decrease on tubular cell damage induced by FA insult (Figs 1C–E and 2A,B). This observation could be explained by an effect of CtsD inhibition on inflammation^18^ or on epithelial tubular cell death^11^. Gene expression of AKI inflammatory mediators was analysed. CXCL-1, CXCL-2, IL-1β, IL-6, TNF-α and RANTES expression was significantly increased in the FA group however, Pepstatin A only significantly decreased IL-1β (Suppl. Fig. S2A–E) but not any other mediators. Neutrophil infiltration into the kidneys caused by FA treatment was also not significantly affected by Pepstatin A treatment (Fig. 2C). We then analysed the effect of Pepstatin A on apoptosis. FA administration led to an increase in cleaved or active caspase-3 in kidney which was reduced by Pepstatin A administration (Fig. 2D). TUNEL staining demonstrated a 50% reduction in the percentage of apoptotic (TUNEL^+^) cells in FA Pepstatin A treated kidneys compared with FA vehicle treated (Fig. 2E). No TUNEL positive cells were detected in the control kidneys. Therefore, CtsD inhibition improves kidney function and reduces apoptosis in a FA induced nephrotoxic AKI model.

### Cathepsin D is upregulated in AKI after ischemia reperfusion injury

**Figure 3:**
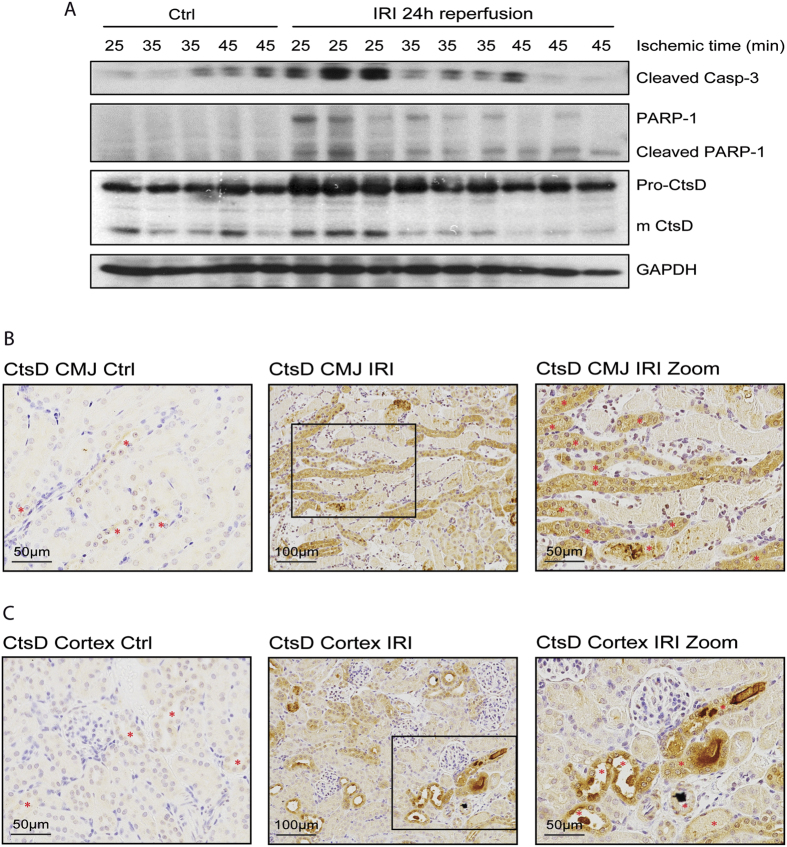
CtsD is increased in IRI induced AKI following a similar trend than pro-apoptotic markers. Western Blot of cleaved caspase-3, PARP-1 and cleaved PARP-1, pro- and mature CtsD and GAPDH **(A)** during increasing ischemic times (25, 35 and 45 minutes) and 24 hours reperfusion in control and IRI kidneys lysates. CtsD representative pictures and magnified area of CMJ or cortex of control kidneys and 25 minutes ischemic 24 hours reperfused kidneys. Red stars * point CtsD expressing cells **(B,C)**. N = 6, t-test, *P ≤ 0.05 or **P ≤ 0.01.

AKI is induced not also by nephrotoxic insults but also by IRI. Both necrosis and apoptosis contribute to tubular loss during IRI^8^. The cell death ratio biases towards necrosis rather than apoptosis as the severity of the damage increases with higher ischemic times^19^. Thus, we first characterized the contribution of apoptosis in a mouse model of renal IRI. We performed different ischemic times (25, 35 and 45 minutes) with the same reperfusion time (24 hours). We analysed two well described apoptotic events, the activation or cleavage of the effector caspase-3 and the inactivation or cleavage of PARP-1^20^. Both, active caspase-3 and caspase dependent cleaved PARP-1 fragment were increased after 25 minutes of ischemia in comparison with the control kidneys (Fig. 3A). However, their expression declined at 35 and 45 minutes, most likely due to a higher contribution to cell death from necrosis rather than apoptosis (Fig. 3A). Of note, active caspase-3 was increased in the right, control kidneys, with longer ischemic times to the left, IRI kidney. This could be explained by the enhanced stress this kidney undergoes due to the longer operative procedure and also by a systemic inflammatory response triggered by increased damage to the IRI kidney. We then characterized CtsD expression in our model. Pro- and mature CtsD followed similar expression pattern than active caspase-3 and cleaved PARP-1 with maximum expression after 25 minutes ischemia and a later decline in expression by 35 and 45 minutes (Fig. 3A). CtsD expression was confirmed in control and 25 minutes IRI kidney tissues. In agreement with the WB, control kidneys showed less CtsD expression which was limited to a fine dotty pattern in some tubular cells (Fig. 3B,C, Suppl. Fig. S3A,B). However, after the ischemic insult CtsD expression increased in epithelial tubular cells from the corticomedullary junction and the cortex (Fig. 3B,C, Suppl. Fig. S3A,B). Interestingly, CtsD was predominately detected in damaged tubules characterized by tubular dilation and the presence of granular casts. In agreement with the FA model (Suppl. Fig. S1A,B), dual immunofluorescence for markers of proximal or distal tubular epithelial cells and CtsD, showed similar results with CtsD mainly localized in distal tubular cells however, some expression was also detected in proximal tubular cells in sham and IRI kidney tissues (Suppl. Fig. S3A,B). Therefore CtsD expression correlates with apoptosis in damaged epithelial tubular cells during IRI.

### Cathepsin D inhibition reduces tubular cell damage after acute IRI

**Figure 4:**
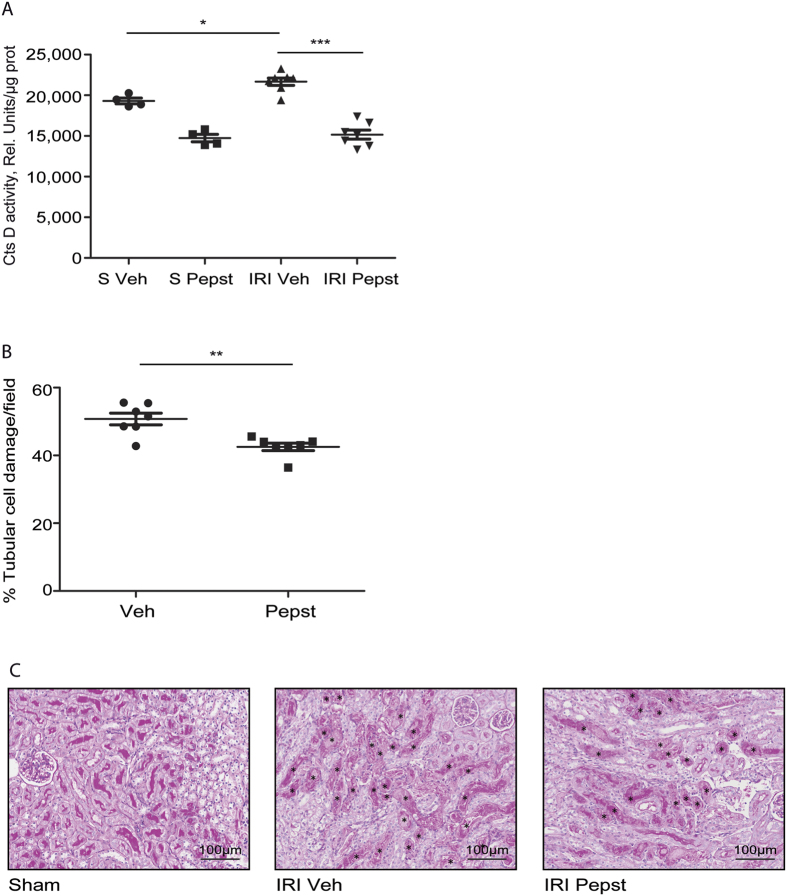
Cathepsin D inhibition reduces tubular cell injury in IRI induced AKI. CtsD fluorometric activity in kidney lysates assessed by the cleavage of a specific fluorescently labelled substrate **(A)** in sham and IRI vehicle or Pepstatin A treated animals. Percentage of tubular cell injury as assessed by tubular dilatation and granular cast formation in CMJ **(B)** of IRI vehicle or Pepstatin A treated kidneys. Representative PAS pictures, damaged cells pointed with a * **(C)** of sham or IRI vehicle or Pepstatin A treated kidneys. Ischemia was performed for 25 minutes and kidneys were reperfused for 24 hours. Animals were treated with vehicle or Pepstatin A 10 mg/Kg 1 hour before surgery and 4 hours post-surgery. N = 7, 1 way ANOVA or t-test,*P ≤ 0.05 or **P ≤ 0.01.

Cathepsin D is known to play a pro-apoptotic role in different cell types^11^, however, its role in IRI induced AKI is unknown. The occurrence of IRI induced AKI can be predicted in some cases as occurs during renal transplant^21^ or major surgery^22^. In that particular cases pre-treatment before the ischemic episode takes place has potential therapeutic application. In order to simulate this kind of scenario we pre-treated the mice with CtsD inhibitor (Pepstatin A) 1 hour before ischemia and 4 hours post-ischemia. We performed 25 minutes ischemia and 24 hours reperfusion, conditions where both apoptosis and CtsD protein levels were higher in our model (Fig. 3A). In agreement with Fig. 3A, CtsD activity was significantly increased after IRI in comparison with sham kidneys (Fig. 4A). Pepstatin A reduced CtsD activity not only in IRI kidneys but also in sham (Fig. 4A). Pepstatin A significantly reduced the percentage of damaged tubular cells in the CMJ (Fig. 4B,C), the part of the kidney most susceptible to injury in this model^21^,^23^. Thus, inhibition of CtsD by Pepstatin A during IRI leads to a reduction of the number of damaged tubular cells in the CMJ.

### Pepstatin A does not affect inflammation but reduces apoptosis after acute IRI

**Figure 5:**
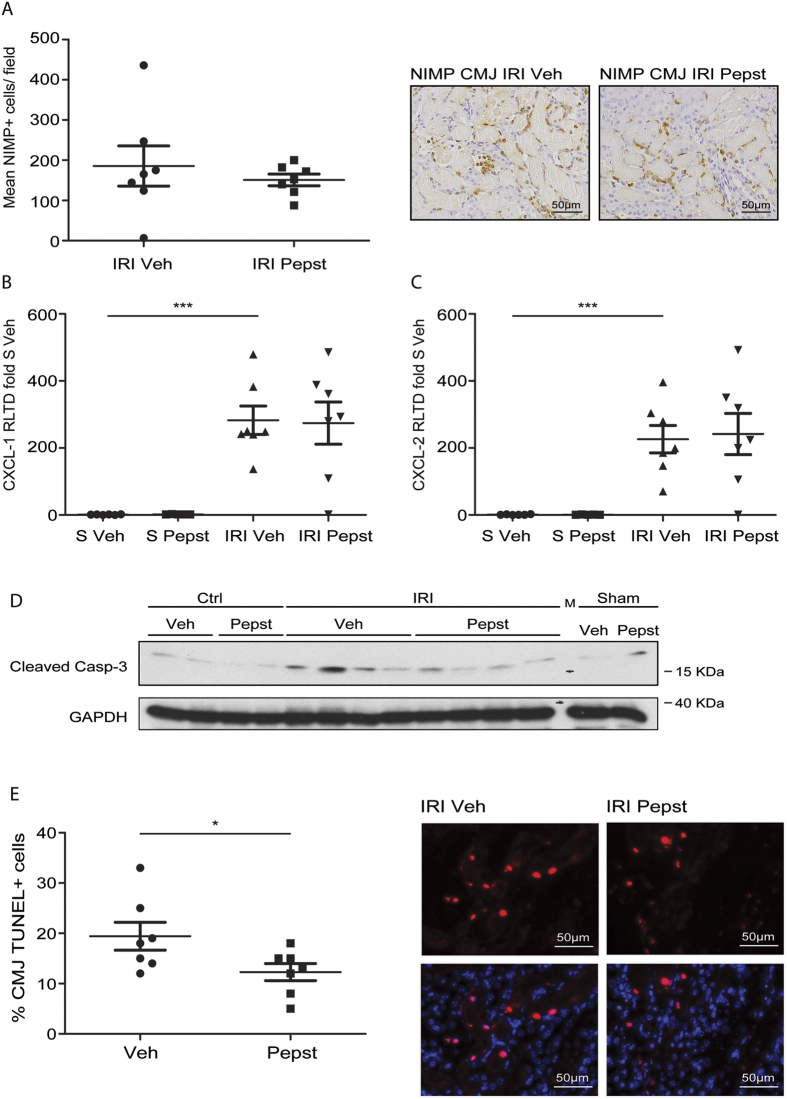
Pepstatin A reduces apoptosis with no alteration on neutrophil infiltration in IRI induced AKI. Average number of NIMP^+^ cells per field with representative pictures in the CMJ of IRI vehicle or Pepstatin A treated kidneys. **(A)** CXCL-1 and CXCL-2 mRNA expression from sham and IRI vehicle or Pepstatin A treated kidneys. **(B,C)** Cleaved caspase-3 and GAPDH WB **(D)** of sham, control or IRI vehicle or Pepstatin A treated kidneys. Percentage of CMJ TUNEL positive cells versus total cells and representative TUNEL only or DAPI merged pictures **(E)** in IRI vehicle or Pepstatin A treated kidneys. Ischemia was performed for 25 minutes and kidneys were reperfused for 24 hours. Animals were treated with vehicle or Pepstatin A 10 mg/Kg 1 hour before surgery and 4 hours post-surgery. N = 7, 1 way ANOVA or t-test,*P ≤ 0.05 or **P ≤ 0.01.

During the reperfusion phase of IRI there is an inflammatory response which is driven by neutrophils amongst other inflammatory cells. CtsD is known to drive apoptosis in neutrophils through activation of caspase-8^13^, therefore CtsD inhibition could lead to a sustained inflammatory response. Thus we analysed the effect of Pepstatin A administration on inflammation in our model. Pepstatin A did not have any effect in the number of neutrophils observed in the CMJ after IRI (Fig. 5A). Gene expression of inflammatory mediators implicated in AKI such as CXCL-1, CXCL-2, IL-1β, IL-6, TNF-α and RANTES were not significantly altered by Pepstatin A treatment (Fig. 5B,C and Suppl. Fig. S4A–D), despite being significantly increased upon IRI. We then analysed whether Pepstatin A was affecting apoptosis induced by IRI. Western blot for active caspase-3 confirmed an increase in apoptosis in IRI kidneys in comparison with control and sham kidneys as well as a decrease with Pepstatin A treatment (Fig. 5D). TUNEL staining in IRI tissues showed a 40% reduction in the percentage of apoptotic cells in the CMJ of IRI Pepstatin A treated animals (Fig. 5E). No TUNEL positive cells were detected in the control kidneys. Thus, Pepstatin A reduces tubular damage and apoptosis with no affectation of the inflammatory response.

### CtsD inhibition reduces hypoxic induced cell death in tubular epithelial cells

**Figure 6:**
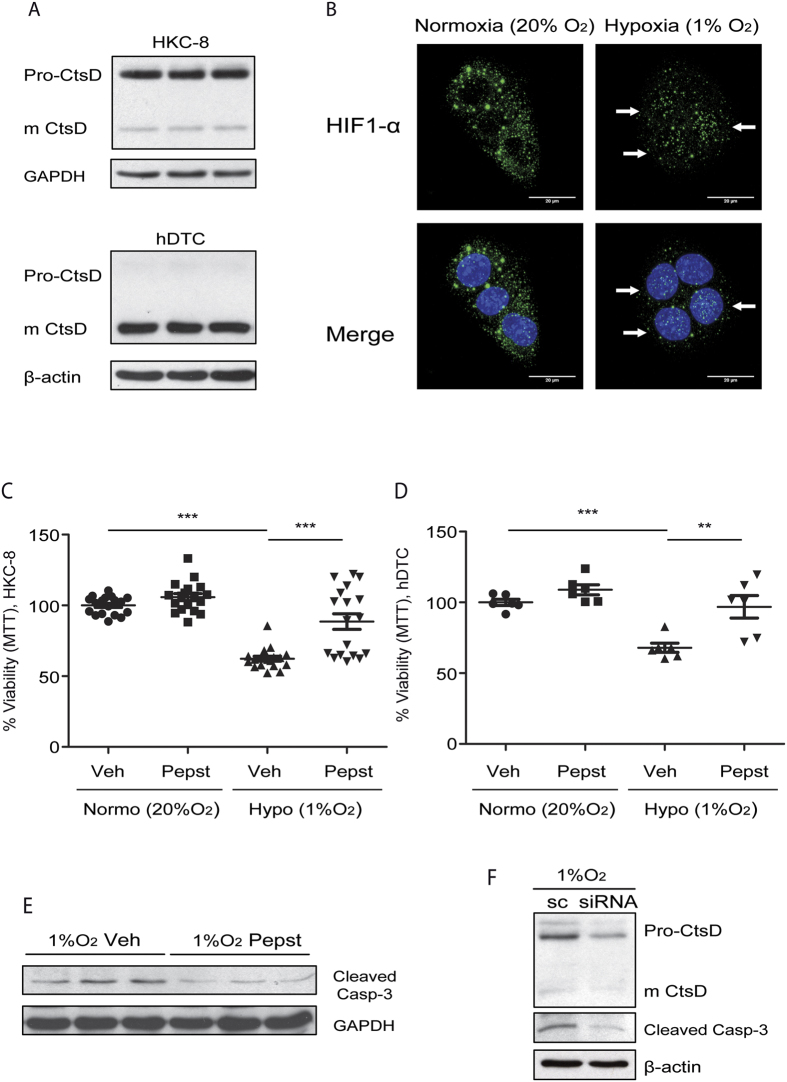
Pepstatin A reduces hypoxic induced apoptosis in tubular epithelial cells. CtsD and GAPDH WB of HKC-8 cells and CtsD and β-actin WB of hDTC. **(A)** HIF-1α immunostaining in HKC-8 cells under normoxic (20% O_2_/5% CO_2_) or hypoxic (1% O_2_/5% CO_2_) conditions for 48 hours. **(B)** White arrows point to HIF-1α located within the nuclei. Percentage of metabolically active viable cells assessed by MTT assay in HKC-8 cells **(C)** or hDTC passage 2 **(D)** treated with vehicle or Pepstatin A under normoxic (20% O_2_/5% CO_2_) or hypoxic (1% O_2_/5% CO_2_) conditions for 48 hours. Cleaved caspase-3 and GAPDH WB in HKC-8 cells under hypoxic conditions for 48 hours treated with vehicle or Pepstatin A. **(E)** Cleaved caspase-3 and β-actin WB in HKC-8 cells under hypoxic conditions for 48 hours treated with scramble or siRNA against CtsD. **(F)** N = 3, 1 way ANOVA,*P ≤ 0.05 or **P ≤ 0.01.

During IRI tubular epithelial cells undergo hypoxia due to a decrease in oxygen supply^23^. CtsD basal levels of expression was first determined in proximal tubular epithelial cell line (HKC-8) and primary human distal tubular cells (hDTC). CtsD level of expression was similar between both cells types, however, the predominant protein form was different with CtsD being mainly expressed as pro-form in HKC-8 cells and active form in hDTC (Fig. 6A). Hypoxic conditions were replicated *in vitro* by culturing HKC-8 and hDTC in 1% oxygen. Hypoxia was confirmed by HIF-1α nuclear translocation (Fig. 6B). Cell viability was assessed in vehicle or Pepstatin A treated cells cultured in 20% or 1% O_2_ concentration. Hypoxia significantly reduced the number of metabolically active viable HKC-8 or hDTC in comparison with cells cultured in normoxia (Fig. 6C,D). Pepstatin A treatment significantly improved cell viability under hypoxic conditions in both cell types (Fig. 6C,D). Thus CtsD play a role in both cell types, however it is likely that CtsD might have a more rapid response in hDTC than in HKC-8, as it is already expressed as active form, while in HKC-8 CtsD pro-form will need to be processed and activated. Active caspase-3 was also reduced when hypoxic cells were treated with Pepstatin A or siRNA against CtsD (Fig. 6E,F). Our *in vitro* results support our *in vivo* IRI findings that inhibition of CtsD with Pepstatin A reduces tubular cell hypoxia-induced apoptotic death.

### Early cathepsin D inhibition protects against fibrosis after IRI induced AKI

**Figure 7:**
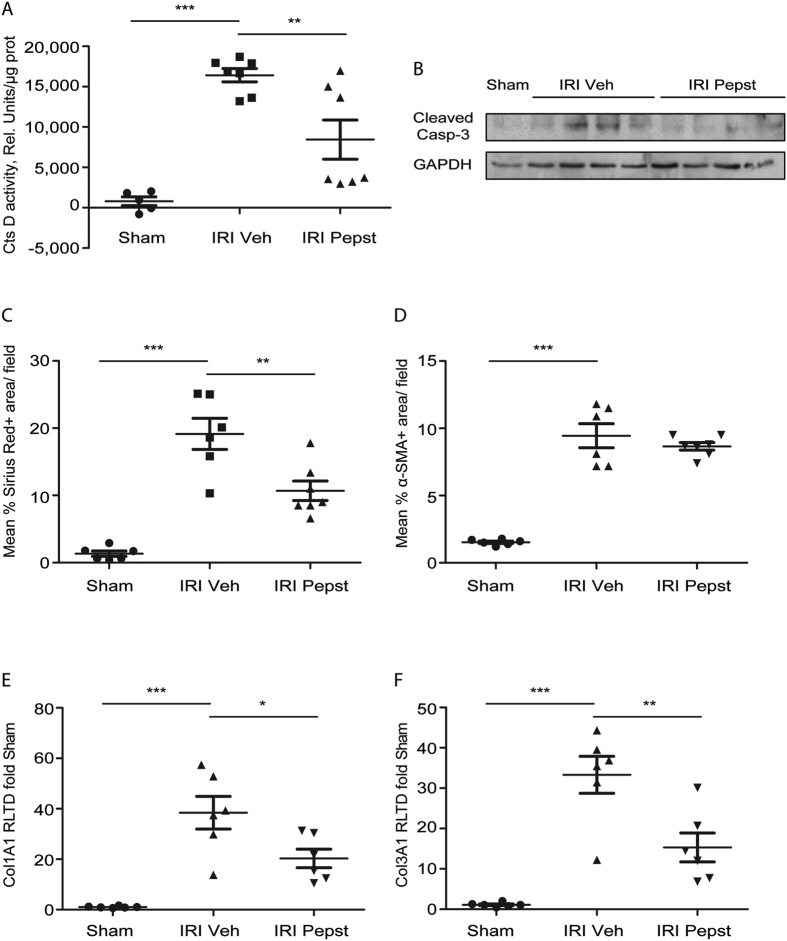
Pepstatin A pre-treatment reduces interstitial fibrosis development from IRI induced AKI. CtsD fluorometric activity in kidney lysates assessed by the cleavage of a specific fluorescently labelled substrate. **(A)** Cleaved caspase-3 and GAPDH WB **(B)** in sham and IRI vehicle or Pepstatin A treated animals. Morphometric analysis of SR^+^ area/field **(C)** or α-SMA^+^ area/field **(D)** of kidney cortex from sham and IRI vehicle or Pepstatin A treated kidneys. Col1A1 **(E)** and Col3A1 **(F)** mRNA expression from sham and IRI vehicle or Pepstatin A treated kidneys. Ischemia was performed for 35 minutes and kidneys were reperfused for 28 days. Animals were treated with vehicle or Pepstatin A 20 mg/Kg 1 hour before surgery and from day 2 post-surgery three times a week up to 28 days. N = 6, 1 way ANOVA or t-test,*P ≤ 0.05 or **P ≤ 0.01.

AKI predisposes to the development of chronic kidney disease (CKD)^24^. During AKI a normal repair response restores the normal tubular epithelium. However, an abnormal repair response (incomplete tubular repair, persistent inflammation, fibroblasts proliferation and excessive extracellular matrix deposition) leads instead to CKD^5^. To analyse whether CtsD inhibition could have an effect on tubulointerstitial fibrosis, Pepstatin A was administered pre- and post-ischemia up to 28 days of reperfusion. IRI kidneys showed a significant increase in CtsD activity in comparison with sham, which was significantly reduced by Pepstatin A treatment (Fig. 7A). As with IRI induced AKI model (Fig. 5D), Pepstatin A also reduced active caspase-3 in kidney at 28 days IRI (Fig. 7B), pointing towards a reduction in apoptosis. Interstitial collagen in IRI kidneys, as assessed by Sirius Red staining, was significantly reduced by Pepstatin A compared to vehicle treatment (Fig. 7C). α-SMA staining, as a marker of interstitial myofibroblasts, was not affected by Pepstatin A treatment (Fig. 7D). Collagen synthesis determined by collagen 1A1 and collagen 3A1 gene expression was significantly decreased (Fig. 7E,F) in IRI Pepstatin A treated kidneys. Therefore, early treatment with Pepstatin A slowed fibrosis development as a consequence of ischemic AKI.

### CtsD expression is increased during acute tubular necrosis (ATN) in transplanted kidneys

**Figure 8:**
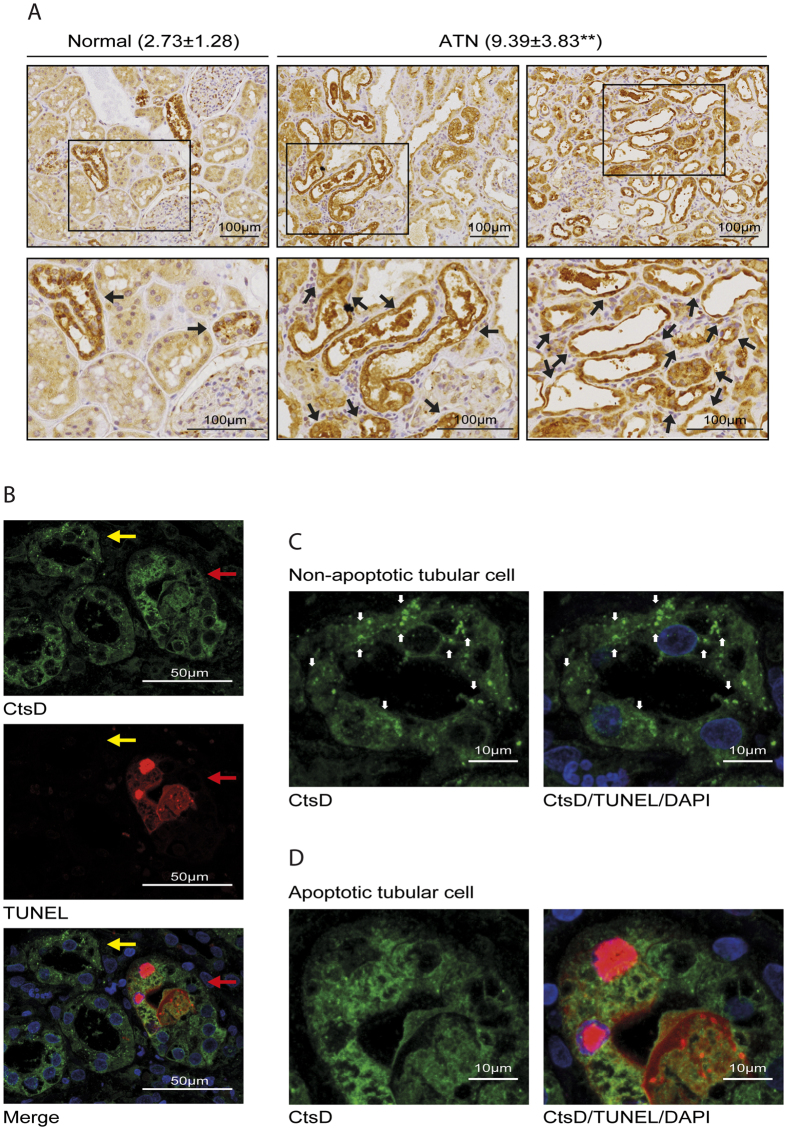
CtsD is highly expressed in human ATN transplant biopsies. Representative pictures of CtsD staining in human normal kidney and ATN transplant kidney biopsies. Percentage of CtsD positive area/total area is expressed as average ± SD. Black arrows point at CtsD expressing tubular cells. **(A)** CtsD/TUNEL dual staining in ATN transplant human kidney biopsy. Yellow arrow points to non-apoptotic and red arrow to apoptotic tubular cells. **(B)** Detail of CtsD distribution in a non-apoptotic tubular epithelial cell (TUNEL^−^/CtsD^+^), white arrows point to vesicular distribution. **(C)** Detail of CtsD distribution in an apoptotic tubular epithelial cell (TUNEL^+^/CtsD^+^). **(D)** A minimum of 6 different normal human kidneys and 9 different transplant ATN kidney biopsies were stained. t-test,*P ≤ 0.05 or **P ≤ 0.01.

We have described CtsD inhibition by Pepstatin A as a therapeutic intervention to reduce apoptosis, tubular damage and improve kidney function in mouse AKI. Pepstatin A administration also reduced subsequent progression from ischemic AKI to interstitial fibrosis. In order to validate some of our findings in human disease we performed CtsD staining in normal human kidney and transplant biopsies demonstrating Acute Tubular Necrosis (ATN). CtsD expression in normal human kidney was mainly detected in distal tubular epithelial cells and some podocytes as assessed by a renal pathologist (KMW) and in agreement with previous reports^25^,^26^ (Fig. 8A). CtsD expression in ATN patients was significantly higher (9.39 ± 3.83) than in normal human kidneys (2.73 ± 1.28) analysed by % of CtsD positive area versus total area with p value = 0.001 (Fig. 8A). In addition, CtsD expression appeared to correlate with the degree of tubular damage in ATN patients. Thus, injured tubular cells characterised by loss of brush border, granular cast formation, tubular dilatation and epithelial cell vacuolization had higher CtsD expression than less damaged cells (Fig. 8A). However, we cannot be certain which is the cell type expressing CtsD in transplant ATN biopsies. Differentiation between proximal and distal tubular cells was not possible due to the loss of their typical morphology as result of severe tubular damage. During apoptosis CtsD is release from lysosomes into the cytosol due to lysosomal membrane permeabilization (LMP), where it can play an active role in apoptosis^11^. To further study CtsD expression during cell death in ATN patients, CtsD was co-stained with TUNEL. During ATN, CtsD was expressed in non-apoptotic (TUNEL^−^) and apoptotic (TUNEL^+^) epithelial tubular cells (Fig. 8B). However, its cellular distribution changed depending on whether cells were undergoing apoptosis. While CtsD was distributed within vacuoles, most likely lysosomes, in non-apoptotic tubular epithelial cells (Fig. 8C), in apoptotic cells CtsD was evident in the cytosol (Fig. 8D). This observation suggests translocation of CtsD from the lysosome into the cytosol during apoptosis in human ATN. Our findings support a possible role for CtsD during tubular epithelial cell death in transplant kidneys with ATN.

## Discussion

The incidence of AKI has risen over the last decades due to the aging population and higher comorbidity associated in this patient group^7^. AKI is often under-recognised, but is associated with elevated risk of early and long-term adverse outcomes^4^. Despite the progress in the management of AKI its mortality rate over the last 50 years remains unchanged at around 50%^27^. AKI also contributes to delayed graft function (DGF) and graft lost in transplantation^21^. Finally, incomplete recovery from AKI can lead to the development of CKD^24^. Therefore there is an urgent need for specific therapies to treat AKI. Thus better knowledge about the cellular mechanisms driving AKI is crucial in order to find new therapeutic candidates.

AKI is a complex disease which can be caused by a variety of insults. However, tubular epithelial cell injury and death with loss of kidney function is common to all. Lysosomal proteases such as CtsD can play multiple roles in apoptosis by degrading different substrates and/or contributing to mitochondrial destabilization^11^. A urinary proteomic analysis identified CtsD as a possible novel prognostic marker for AKI. In this study IGFBP-7 and CtsD were validated by proteomics and ELISA as differentially regulated in urine from late/non-recovery compared with early recovery AKI patients^28^,^29^. Despite all these evidences indicating a possible role of CtsD in cell death during AKI, its contribution is still unknown.

Here we describe an increase of CtsD expression in two different models of AKI, nephrotoxic and ischemic induced (Figs 1A and 3A, Suppl. Figs S1A,B and S3A,B). CtsD was highly expressed in damaged tubular cells during both types of injury in comparison with control kidneys, pointing towards a possible contribution of CtsD to cell injury during AKI (Figs 1B and 3B,C). Interestingly, dual immunofluorescence for proximal or distal tubular cell markers and CtsD, reveal CtsD expression mainly in distal tubular cells, although some expression was also detected in proximal tubular cells, in both normal and FA or IRI injured kidneys (Suppl. Figs S1A,B and S3A,B) pointing towards a possible cell specific function for CtsD. Further investigation will need to be done in the future to clarify the physiological relevance of this differential distribution.

In agreement with our findings in mouse AKI, CtsD staining in human transplant biopsies with ATN confirmed high levels of CtsD expression in damaged tubular epithelial cells in comparison to normal kidney tissues (Fig. 8A). During apoptosis, lysosomal membrane permeabilization allows translocation of CtsD from the lysosome into the cytosol^9^, where it exerts its pro-apoptotic function. Microinjection of CtsD into the cytosol is sufficient to trigger mitochondrial permeabilization and apoptosis, which is prevented by a caspase-3 or CtsD (Pepstatin A) inhibitors^30^. When analysing the cellular localization of CtsD in human ATN there was stronger cytosolic staining in cells undergoing apoptosis whereas in non-apoptotic cells a more vesicular, most likely lysosomal, pattern was evident (Fig. 8B–D).

CtsD optimal activity occurs in the acidic pH found within the lysosomes. Although CtsD is still active at cytosolic neutral pH, its life-time is limited due to reversible deprotonation of the active aspartate site^11^. However, there is several mechanisms that might contribute to prolonged CtsD activity during apoptosis, such as cytosolic acidification^31^ or substrate binding. CtsD activity was confirmed in our studies showing a significant increase during injury (Figs 4A and 7A).

To further analyse the role of CtsD during cell death in AKI we used the CtsD inhibitor, Pepstatin A. Pepstatin A is the best available inhibitor against CtsD however, it can affect other proteases of the aspartic endopeptidase A1 family. Most of the proteases of the A1 family are specifically expressed in other organs, such as stomach (Pepsin and CtsE) or central nervous system (BACE-1 and -2). Although renin is expressed in the kidney, Pepstatin A is a weak renin inhibitor, to the extent that 26.000 times more Pepstatin A is required to inhibit renin (Ki = 13000 μmol/L) to a similar level than CtsD (Ki = 0.5 μmol/L)^32^. Nevertheless we cannot exclude additional effects of Pepstatin A on other A1 peptidases in our studies, we consider its action on CtsD as likely having the dominant effect. CtsD knock-out mice have massive neuronal cell death and die approximately 6 weeks after birth due to neurological disorders^33^, replicating human deficiency^34^,^35^. Pepstatin A dose for our studies was more than 50 times below the previously described IC_50_ for mice^36^, at this dose Pepstatin A reduced CtsD activity without complete inhibition (Figs 4A and 7A), which may have led to undesirable secondary effects.

We first analysed the effect of CtsD inhibition on inflammation as cathepsins play an important role in the immune response^18^, modulating tissue damage and cell death. Specifically CtsD plays an important role driving neutrophil apoptosis by directly activating the initiator caspase-8. CtsD deficiency leads to delayed neutrophil apoptosis and an amplified and prolonged innate immune response^13^. CtsD inhibition did not induce any changes in neutrophil infiltration in FA or IRI induced AKI (Figs 2C and 5A). Expression of inflammatory genes implicated in the development of AKI was also not significantly altered by Pepstatin A treatment (Suppl. Fig. S2A–E, Fig. 5B,C, Suppl. Fig. S4A–D). Although this does not prove that Pepstatin A did not alter the inflammatory response in these models it would suggest that this was not a major effect.

Administration of Pepstatin A significantly improved kidney function (Fig. 1C–E) in FA nephrotoxic induced AKI model and reduced apoptotic tubular cell death in both models (Figs 2D–E and 5D–E), showing an overall reduction in the degree of tubular damage (Figs 2A,B and 4B,C). The effect of CtsD inhibition on cell viability was confirmed *in vitro* in a human proximal tubular epithelial cell line, HKC-8, and human primary distal tubular epithelial cells (hDTC)^26^ under hypoxic conditions (1%O_2_) using Pepstatin A. CtsD inhibition significantly rescued the reduction in cell viability caused by hypoxic conditions (Fig. 6C,D). A decrease in apoptosis was confirmed under the same conditions (1%O_2_) using Pepstatin A and specific siRNA against CtsD (Fig. 6E,F). It is therefore possible that Pepstatin A reduced functional and histological tubular injury by reducing apoptotic cell death. Proving a causal link between reduced apoptosis and favourable renal outcomes is difficult *in vivo*, but the cell protective effects of Pepstatin A are supported by the *in vitro* data presented.

AKI can contribute or exacerbate the progression of CKD due to an abnormal or incomplete repair response. We have previously shown that Pepstatin A treatment from day 5 after IRI results in reduction of renal fibrosis due to an increase in collagen degradation with no affectation of collagen gene transcription^26^. In this new model, Pepstatin A pre-treatment before IRI seemed to have additional beneficial effect over the development of the injury. As well as a decrease in interstitial collagen (Fig. 7C), we also observed a decrease in apoptosis (Fig. 7B) and a reduction in the expression of pro-fibrotic genes (Fig. 7E,F). We propose that CtsD can influence disease progression by a dual mechanism of action contributing to apoptosis in the acute phase and to collagen turnover during the chronic phase. The protection from both acute injury and subsequent progression to CKD identifies CtsD, and potentially other proteases, as potential therapeutic targets.

In summary, we report CtsD as an important mediator for apoptotic cell death during AKI. Our work has focused on the role of CtsD, however, we cannot discard the participation of other lysosomal proteases such as CtsB or L during AKI and further investigation will be needed to clarify their roles. New therapies to reduce apoptotic cell death during AKI are already under study (caspase inhibitors^37^, p53 inhibitors^38^ and PARP inhibitors^39^). Pepstatin A have been safely used in clinical trials for duodenal ulcer^40^,^41^ however, it presents problems such as poor solubility and low bioavailability. New drugs against CtsD can be developed^42^, allowing more effective and better targeting of CtsD. Our work opens new and exciting prospects for the treatment of AKI by inhibiting lysosomal protease induced apoptosis.

## Methods

### Induction of acute kidney injury in mice

All animal experimental protocols and studies were done in accordance to the UK Home Office regulation and where approved by UK Home Office with project licence 60/4521. Animal models were performed in 8–10 week C57BL/6 females.

For folic acid (FA) induced nephrotoxic injury, single intraperitoneal injection of 250 mg/Kg of FA in 0.3 M NaHCO_3_ or vehicle alone was administered. Vehicle or Pepstatin A (20 mg/Kg) was injected intraperitoneally 45 minutes and 24 h post-FA administration. Animals were culled 48 h after FA injection.

For ischemia reperfusion injury (IRI) left renal pedicle was clamped for 25, 35 or 45 minutes and kidneys allowed to reperfused for 24 h. Contralateral right kidneys (to simplify called controls in the text) and sham kidneys were used as controls. Sham animals underwent a mocked surgical procedure consisting in exactly the same surgical protocol as the animals undergoing IRI apart from the left renal pedicle clamp. Vehicle or Pepstatin A (10 mg/Kg) were administered 1 h before surgery and 4 h post-surgery by intraperitoneal injection. Animals were culled 24 h after surgery. A minimum of 8 or 7 animals were used in each FA or IRI experimental groups respectively.

### Induction of tubulointerstitial fibrosis in mice

In 8–10 week C57BL/6 females the left renal pedicle was clamped for 35 minutes kidneys allowed to reperfused for 28 days. Sham animals underwent a mocked surgical procedure. Vehicle or Pepstatin A (20 mg/Kg) were administered by intraperitoneal injection 1 hour before surgery and from day 2 post-surgery three times a week up to 28 days. A minimum of 6 animals were used in each experimental IRI group.

### Study approval for human samples

Acute Tubular Necrosis (ATN) transplant C4b negative biopsies were taken under full ethical approval and written informed patient consent in accordance to the approved guidelines. Ethical approval was granted by the NRES Committee East Midlands-Derby Research Ethics Committee (REC reference 13/EM/0311).

### Tubular damage assessment

PAS was performed in 4 μm kidney sections following standard procedures. Tubular damage was assessed in 10–20 random 200X fields in the cortex for the FA injury or the CMJ for the IRI. Damaged tubules were classified according to previously described AKI clinical pathological description^43^ using the following criteria: tubular dilation, loss of brush border and epithelial flattening for the FA model and tubular dilation and granular cast formation for the IRI model. Results were expressed as percentage of the number of damaged cells versus total number of cells per field.

### Biochemical analysis

Blood urea nitrogen (BUN) and serum creatinine assays were performed in the Clinical Biochemistry department at the Royal Victoria Infirmary, Newcastle.

### KIM-1 ELISA

KIM-1 ELISA was performed in mouse urine samples using mouse TIM-1/KIM-1/HAVCR Quantikine ELISA Kit from R&D according to manufacturer’s instructions.

### Cell culture

HKC-8 cells^44^ or primary human distal tubular epithelial cells (hDTC) were cultured in 1:1 Dulbecco’s modified Eagle’s: F12 medium or DMEM supplemented with 100 U/ml penicillin, 100 μg/ml streptomycin, 2 mM L-glutamine, 5% FBS, and maintained at 37 °C at an atmosphere of 20% O_2_/5% CO_2_ (normoxia) or 1% O_2_/5% CO_2_ (hypoxia).

### Isolation of human primary distal tubular cells (hDTC)

Human kidneys cells were isolated from adult kidneys after surgical resection in accordance to the Research Ethics Committee guidelines and ethical approval granted by the NRES Committee East Midlands-Derby (REC reference 13/EM/0311), subject to written informed patient consent. Human distal tubular cell isolation and characterization was performed as previously described^26^.

### siRNA transfection

HKC-8 were transfected with 50 nM Scramble or 50 nM CtsD siRNA (St Cruz Biotechnology) using INTERFERin (Polyplus) according to manufacturer’s instructions for 48 hrs.

### Cell viability assay (MTT)

HKC-8 cells or passage 2 hDTC were seeded at 60–70% confluence and treated ± Pepstatin A 10 mg/mL for 48 h under normoxia (20% O_2_/5% CO_2)_ or hypoxia (1% O_2_/5% CO_2_). MTT was performed according to standard procedure^45^. Data is expressed as the difference between 570–630 nm.

### CtsD activity assay

Cathepsin D activity was determined using Cathepsin D activity assay kit from Abcam as previously described^26^. Results are expressed as a slope of fluorescence emission after 1 h per μg of protein.

### Immunohistochemistry in mouse and human samples

Formalin-fixed 4 μm kidney sections were stained as previously described: α-SMA (Sigma) and CtsD (St Cruz Biotechnolgy)^26^ or NIMP (Abcam)^46^. Image analysis was performed in a minimum of 10 random 200X fields with a Nikon Eclipse Upright microscope. Using NIS-Elements BR Analysis software, thresholds for NIMP positive cells or for percentage of α-SMA positive area were created and the analysis was run automatically. For CtsD human staining, sections were assessed by an expert histopathologist (KMW). CtsD quantification was performed applying the same automatic threshold for the maximum pixel intensity to all the images using NIS-Elements BR Analysis software. The totality of the ATN biopsy (up to 10 random 100X fields) and 10 random 100X fields for normal human kidneys were quantified. Data is expressed as the average of the % positive area/total area. A minimum of 6 different normal human kidneys and 9 different patient biopsies were stained.

### Sirius Red staining

Paraffin embedded 4 μm kidney sections were routinely stained with 0.1% Sirius Red following standard procedures. For Sirius Red image analysis was performed as described for α-SMA in the immunohistochemistry section.

### TUNEL staining in kidney slides

TUNEL was performed in 4 μm paraffin embedded kidney sections according to manufacturer’s instructions using Roche *in situ* Cell Death Detection Kit, TMR. Sections were mounted using DAPI conjugated mounting medium. Analysis was performed in a Zeiss Axio fluorescent microscope. TUNEL or DAPI positive nuclei were quantified in 10–18 random 200X fields per section. Results were expressed as percentage of the number of positive TUNEL nuclei versus total nuclei (DAPI).

For dual TUNEL/CtsD immunofluorescence staining, microwave citrate saline antigen retrieval followed by 0.25% Triton X-100 permeabilization was performed. Sections were then blocked and anti-CtsD antibody was added in 1% BSA overnight. After washing, anti-goat-FITC secondary antibody and Tunnel Reaction Mixture solution were applied sequentially for 1 h each according to manufacturer’s instructions. Sections were mounted with ProLong^®^ Diamond DAPi conjugated mounting medium and analysed using a Nikon A1 confocal microscope. Pictures were taken sequentially at 600X oil. 22 images of 0.3 μm slice were acquired per slide. Image analysis was performed using Image J software. Images are expressed as merged channels, being each channel the maximum intensity Z stack projection of the 22 individual steps.

### Dual immunofluorescence in kidney tissue

4 μm formalin-fixed kidney tissue sections were deparaffinised and heat mediated citrate saline antigen retrieval was performed. After permeabilization with 0.25% Triton X-100, sections were blocked with 5% BSA and primary antibodies, Thiazide-Sensitive NaCl Cotransporter (NCC) (Millipore) or Aquaporin-1 (Abcam) and CtsD (St Cruz Biotechnolgy) applied in 1% BSA overnight. After washing, the secondary antibodies were sequentially added, donkey anti-rabbit Alexa 594 and anti-goat-FITC. An extra blocking step with 20% rabbit serum was added between secondaries. Sections were mounted with ProLong^®^ Diamond DAPi conjugated mounting medium (Invitrogen) and analysed using a Nikon A1 confocal microscope. Pictures were taken sequentially at 600X oil and 1.75X electronic zoom was made of a region of interest. 8 images of 0.75 μm slice were acquired per slide. Image analysis was performed using Image J software. Images are displayed as the maximum intensity Z stack projection of the 8 individual steps of each channel.

### Immunocytochemistry in epithelial tubular cells

HKC-8 were cultured in glass coverslips, fixed with formalin and permeabilized with 0.1% saponine/0.5% BSA. After blocking with 3% BSA, primary antibody anti-HIF-1α was applied for 90 minutes. After washing, secondary anti-mouse-FITC antibody was added and coverslips were mounted with ProLong^®^ Diamond DAPi conjugated mounting medium. The coverslips were analysed using a Nikon A1 confocal microscope. Pictures were taken sequentially at 600X oil. 3X electronic zoom was made of a region of interest. 10–11 images of 0.75 μm slice were acquired per coverslip. Image analysis was performed using Image J software. Images are expressed as merged channels, being each channel the maximum intensity Z stack projection of the 11 individual steps.

### SDS PAGE and immunoblotting

Lysates from kidney or HKC-8 cells were prepared with radioimmune precipitation assay buffer (RIPA). Proteins (10–30 μg) were resolved in 8–15% SDS-PAGE and transferred into nitrocellulose membranes. Membranes were blocked and incubated overnight with the primary antibodies anti-Cathepsin D (St Cruz Biotechnology), anti-cleaved caspase-3 (Cell Signaling), anti-PARP (Cell Signaling) and anti-GAPDH (Abcam). After TBS-Tween washing and incubation with the appropriate HRP conjugated secondary antibody, membranes were developed with ECL (Thermo Scientific).

### RNA Isolation and Real Time PCR

Kidney RNA was extracted and cDNA was synthetized as previously described^26^. Real time PCR was performed with SYBR Green JumpStart ready mix according to manufacturer’s instructions. Data was calculated using ∆∆Ct and 18S was used as a housekeeping gene. Data is plotted against vehicle sham or control group. Primer sequences used are reported in the Supplementary Table 1.

### Statistics

Results are expressed as mean ± SEM unless otherwise stated in the figure legend. All p values were calculated using one way ANOVA followed by Bonferroni’s test or two tailed unpaired student’s t-test. *P ≤ 0.05, **P ≤ 0.01 or ***P ≤ 0.001 was considered statistically significant.

## Additional Information

**How to cite this article**: Cocchiaro, P. *et al.* Lysosomal protease cathepsin D; a new driver of apoptosis during acute kidney injury. *Sci. Rep.* **6**, 27112; doi: 10.1038/srep27112 (2016).

## Supplementary Material

Supplementary Information
