# Supplementary material for: Lysosomal protease cathepsin D; a new driver of apoptosis during acute kidney injury
Source: Sci Rep. 2016 Jun 7;6:27112. doi: 10.1038/srep27112 (PMC4895243; doi:10.1038/srep27112)
Supplement: Supplementary Information [file srep27112-s1.pdf]

Lysosomal protease cathepsin D; a new driver of apoptosis during acute kidney injury

Pasquale Cocchiaro[1,2#], Christopher Fox[1#], Nicholas W Tregidgo[1], Rachel Howarth[1], Katrina M Wood[3], Gerhard R Situmorang[1,4], Luigi M Pavone[2], Neil S Sheerin[1], Anna Moles[1*]

[1] Fibrosis Research Group, Institute of Cellular Medicine, Newcastle University, Newcastle Upon Tyne, UK

[2] Department of Molecular Medicine and Medical Biotechnology, University of Naples Federico II, Italy

[3] Department of Cellular Pathology, Royal Victoria Infirmary, Newcastle Upon Tyne, UK

[4] Urology Department, RSCM/Faculty of Medicine, University of Indonesia

[#]Both authors contributed equally

*Corresponding author

Address correspondence to: Anna Moles, Fibrosis Research Group, Institute of Cellular Medicine, Newcastle University. 4th Floor William Leech Building, Medical School, Framlington Place, Newcastle Upon Tyne, NE2 4HH, United Kingdom.

Phone: +44 (0)191 208 3886; E-mail: ana.moles@ncl.ac.uk.

Supplementary Figure S1: Differential expression of CtsD in proximal and distal tubular epithelial cells in vehicle and FA injured kidneys

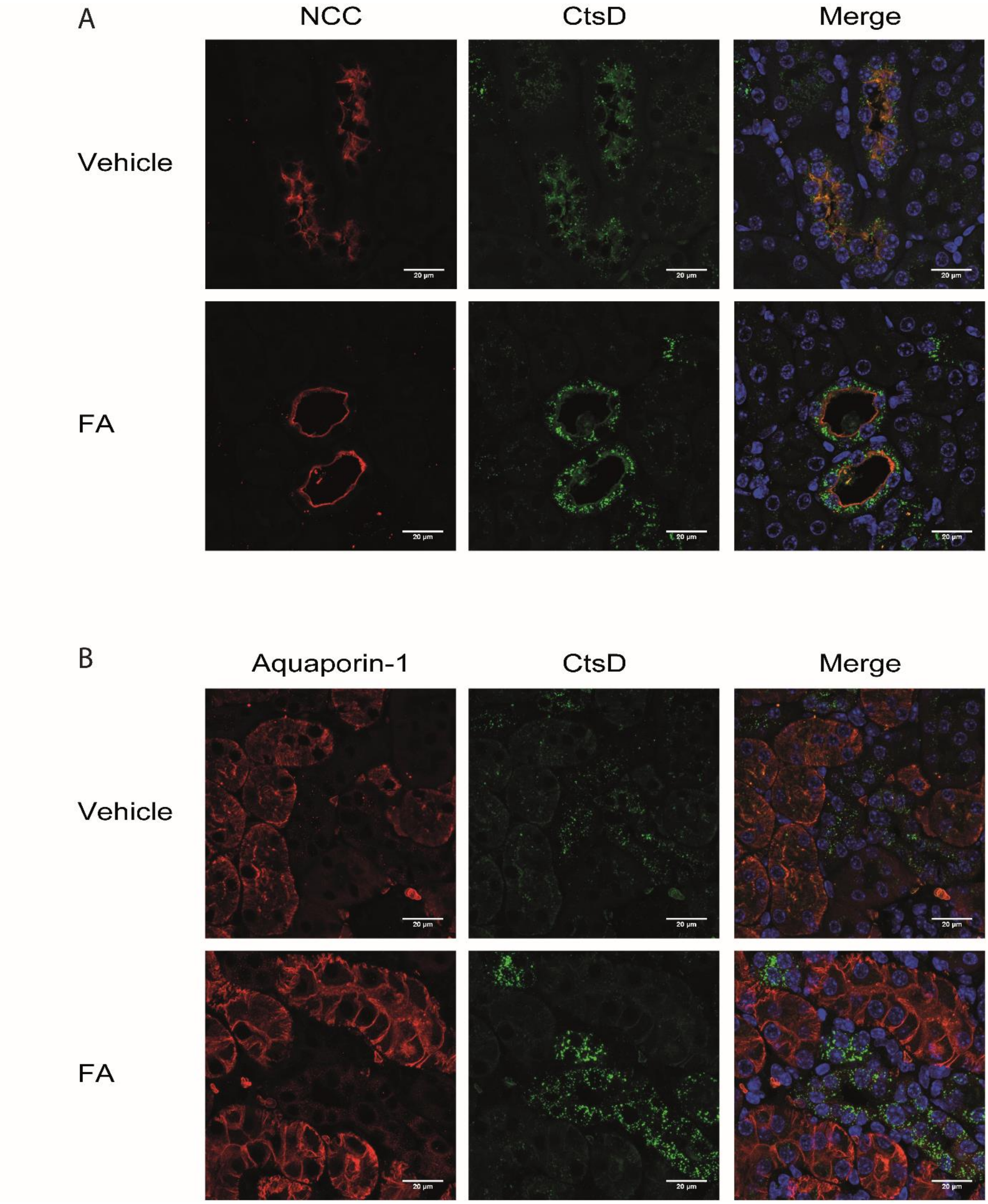

Supplementary Figure S2: Pepstatin A does not affect inflammatory mediators during nephrotoxic induce AKI

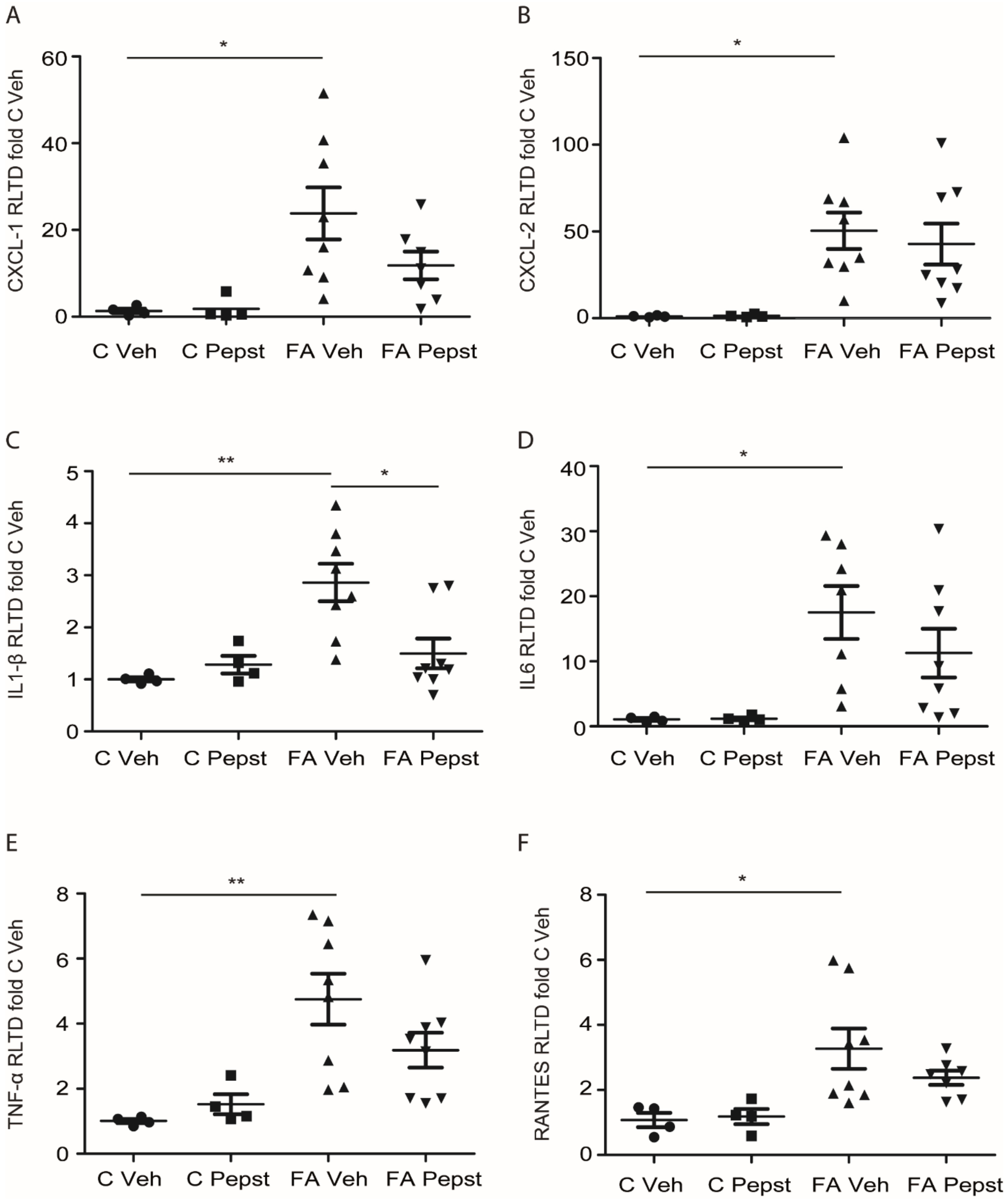

Supplementary Figure S3: Differential expression of CtsD in proximal and distal tubular epithelial cells in sham and IRI injured kidneys

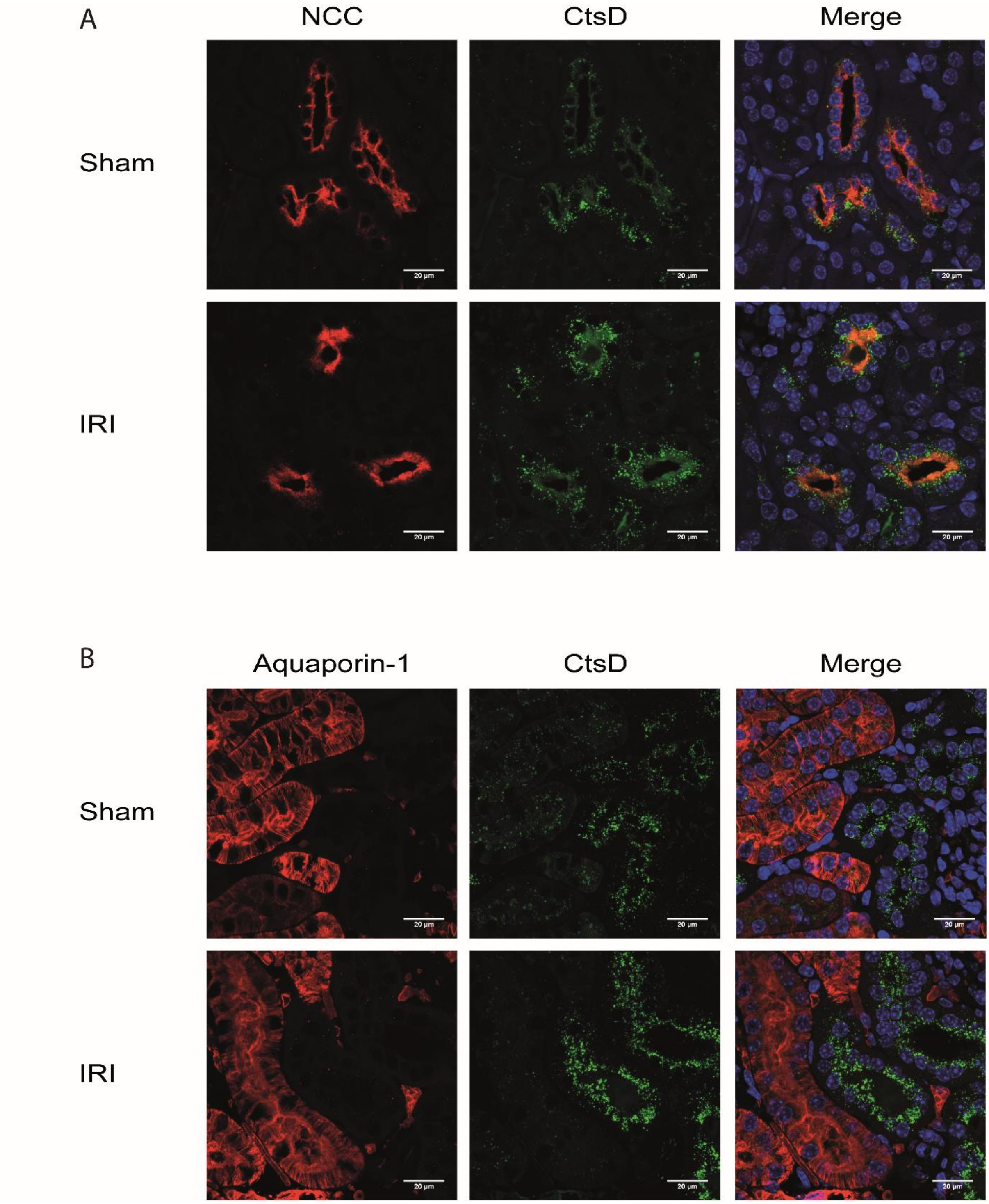

Supplementary Figure S4: Pepstatin A does not affect inflammatory mediators during IRI induce AKI

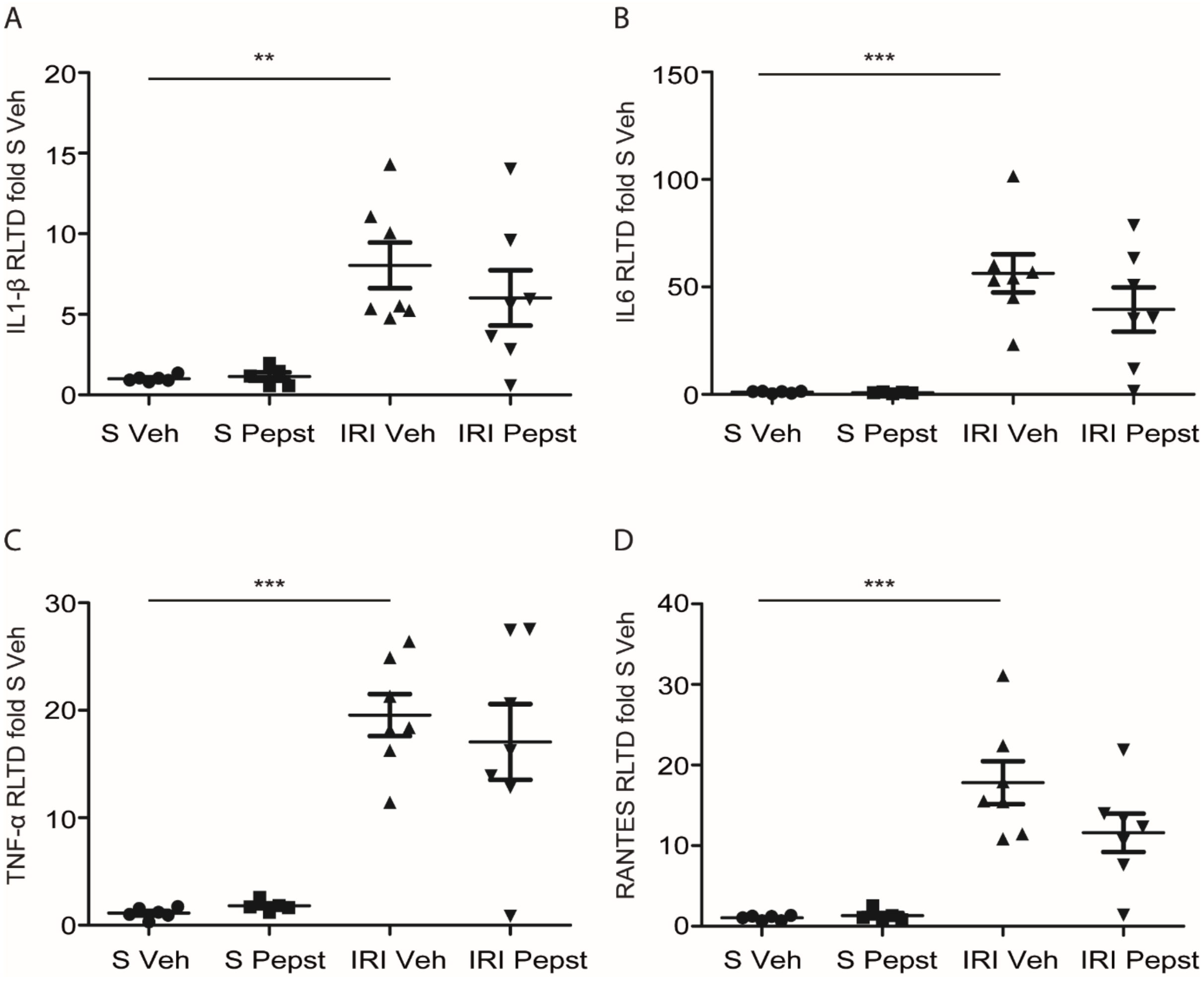

Supplementary Figure S1: Differential expression of CtsD in proximal and distal tubular epithelial cells in vehicle and FA injured kidneys

Representative confocal microscopy pictures of NCC (distal tubular cell)/CtsD **(A)** or Aquaporin-1 (proximal tubular cell)/CtsD **(B)** dual immunofluorescence in vehicle or FA injured kidneys for 48 hours. Pictures are displayed as NCC or Aquaporin-1 (red), CtsD (green) and merge with DAPI (blue) and represent the Z stack projection of the maximum intensity for each of the channels. Scale bar represents 20µm. N=8.

Supplementary Figure S2: Pepstatin A does not affect inflammatory mediators during nephrotoxic induce AKI

CXCL-1 **(A)**, CXCL-2 **(B)**, IL1-β **(C)**, IL-6 **(D)**, TNF-α **(E)** and RANTES **(F)** mRNA expression from control and 48 hours FA vehicle or Pepstatin A treated kidneys. Animals were treated with vehicle or Pepstatin A (20mg/Kg) 45 minutes before and 24 hours post-FA. N=8, 1 way ANOVA, $*P \leq 0.05$ or $**P \leq 0.01$.

Supplementary Figure S3: Differential expression of CtsD in proximal and distal tubular epithelial cells in sham and IRI injured kidneys

Representative confocal microscopy pictures of NCC (distal tubular cell)/CtsD **(A)** or Aquaporin-1 (proximal tubular cell)/CtsD **(B)** dual immunofluorescence in sham or IRI injured kidneys (25 minutes ischemia, 24h reperfusion). Pictures are displayed as NCC or Aquaporin-1 (red), CtsD (green) and merge with DAPI (blue) and represent the Z stack projection of the maximum intensity for each of the channels. Scale bar represents 20µm. N=7.

Supplementary Figure S4: Pepstatin A does not affect inflammatory mediators during IRI induce AKI

IL1-β **(A)**, IL-6 **(B)**, TNF-α **(C)** and RANTES **(D)** mRNA expression from sham and IRI vehicle or Pepstatin A treated kidneys. Ischemia was performed for 25 minutes and kidneys were reperfused for 24 hours. Animals were treated with vehicle or Pepstatin A 10mg/Kg 1 hour before surgery and 4 hours post-surgery. N=7, 1 way ANOVA,*P ≤ 0.05 or **P ≤ 0.01.

Supplementary table 1: Mouse primer sequences

Gene (GenBank Accession)	Primer sequence
Col1A1 (NM_007742)	Fw: 5’-TTCACCTACAGCACGCTTGTG-3’
	Rv: 5’-GATGACTGTCTTGCCCCAAGTT-3’
Col 3A1 (NM_009930)	Fw: 5’-CTGTAACATGGAAACTGGGGAAA-3’
	Rv: 5’-CCATAGCTGAACTGAAAACCACC-3’
CXCL-1 (NM_008176)	Fw: 5’- CTGGGATTCACCTCAAGAACATC-3’
	Rv: 5’- CAGGGTCAAGGCAAGCCTC-3’
CXCL-2 (NM_009140)	Fw: 5’- CCAACCACCAGGCTACAGG-3’
	Rv: 5’- GCGTCACACTCAAGCTCTG-3’
IL-1β (NM_008361)	Fw: 5’-CAACCAACAAGTGATATTCTCCATG-3’
	Rv: 5’-GATCCACACTCTCCAGCTGCA-3’
IL-6 (NM_031168)	Fw: 5’- TAGTCCTTCCTACCCCAATTTCC-3’
	Rv: 5’- TTGGTCCTTAGCCACTCCTTC-3’
RANTES (NM_013653)	Fw: 5’-TGCTGCTTTGCCTACCTCTCC-3’
	Rv: 5’- TGGCACACACTTGGCGGTTCC-3’
TNF-α (NM_013693)	Fw: 5’-CCCTCACACTCAGATCATCTTCT-3’
	Rv: 5’- GCTACGACGTGGGCTACAG-3’
